# The TRPV5/6 calcium channels contain multiple calmodulin binding sites with differential binding properties

**DOI:** 10.1007/s10969-012-9128-4

**Published:** 2012-02-22

**Authors:** Nadezda V. Kovalevskaya, Fedir M. Bokhovchuk, Geerten W. Vuister

**Affiliations:** 1 Department of Protein Biophysics, IMM, Radboud University Nijmegen, Heyendaalseweg 135, 6525 AJ Nijmegen, The Netherlands; 2 Department of Protein Biophysics, NCMLS building, route 260, Geert Grooteplein Zuid 26-28, 6525 AG Nijmegen, The Netherlands; 3 Department of Biochemistry, University of Leicester, Henry Wellcome Building, Lancaster Road, Leicester, LE1 9HN UK

**Keywords:** Calmodulin, Multiple binding, Regulation, TRP channels, TRPV5/6

## Abstract

The epithelial Ca^2+^ channels TRPV5/6 (transient receptor potential vanilloid 5/6) are thoroughly regulated in order to fine-tune the amount of Ca^2+^ reabsorption. Calmodulin has been shown to be involved into calcium-dependent inactivation of TRPV5/6 channels by binding directly to the distal C-terminal fragment of the channels (de Groot et al. in Mol Cell Biol 31:2845–2853, 12). Here, we investigate this binding in detail and find significant differences between TRPV5 and TRPV6. We also identify and characterize in vitro four other CaM binding fragments of TRPV5/6*,* which likely are also involved in TRPV5/6 channel regulation. The five CaM binding sites display diversity in binding modes, binding stoichiometries and binding affinities, which may fine-tune the response of the channels to varying Ca^2+^-concentrations.

**Electronic supplementary material:**

The online version of this article (doi:10.1007/s10969-012-9128-4) contains supplementary material, which is available to authorized users.

## Introduction

The ubiquitous calcium sensor calmodulin (CaM) is known to be a constitutive or dissociable Ca^2+^-sensing subunit for a large variety of ion channels including TRP family channels, various ligand-gated and voltage-gated Ca^2+^ channels [1]. Mounting evidence indicates that channel regulation by calmodulin can involve multiple calmodulin binding sites acting in concert [2], although at present little is known about the functional role of the potential multiple CaM molecules bound to a single functional channel.

One class of well-studied systems are the voltage-gated Ca_v_1-2 channels [3]. These channels contain several CaM binding sites in their C-termini that are involved in the regulation of the channel. The crystal structure of CaM bound to the C-terminal fragment of the Ca_v_1.2 channel has previously been reported [4] and this prompted models for explaining the role of CaM in CDI (calcium-dependent inactivation) and CDF (calcium-dependent facilitation) [5–7]. Subsequently, three additional CaM binding sites have been identified in the N-terminus and in the loop between transmembrane helices I and II [8]. Since their interactions with CaM were characterized only in vitro it is not clear whether these CaM binding sites are relevant in vivo and hence what their functional role could be.

**Fig. 1:**
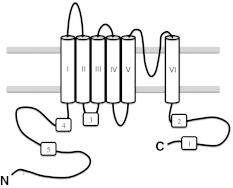
Topology diagram of a typical TRP channel consisting of six transmembrane domains and intracellular N- and C-termini. Predicted CaM binding sites are numbered started from C-terminal side. V5p1 and V6p1 peptides correspond to the CaM binding site 1 of TRPV5 and TRPV6, respectively, as V5p2, V5p3/V6p3, V5p4 and V5p5 correspond to the CaM binding site 2, 3, 4 and 5 of TRPV5/6 (see also Table 1)

In TRP channels, which consist of 6 transmembrane helices and intracellular N- and C-termini (Fig. 1), two CaM binding sites have thus far been identified. However, to date rather limited information is available about the exact role of calmodulin in their regulation [2]. For several TRP channels binding of CaM to the C-terminal intracellular part is reported to be crucial for calcium-dependent inactivation [9, 10], but very few details about the molecular mechanisms are available [11].

In our recent work [12], for the interaction between the distal part of the C-terminus of TRPV5 and CaM, we observed an unexpected 1:2 CaM:TRPV5 binding stoichiometry. We proposed a model which explained the role of CaM binding in calcium-dependent inactivation of the TRPV5 channel. Here, we present the details of the CaM binding to the distal part of the C-terminus of TRPV5 and its close homologue TRPV6. In addition, we also report the in vitro characterization of four other CaM binding sites in TRPV5/6 channels and speculate about their possible functional role.

## Materials and methods

### Peptide synthesis

Peptides corresponding to the amino acid residues 133–154, 310–330, 401–428, 591–612, 696–712 and 696–729 of human TRPV5 and 401–428 and 690–716 of human TRPV6 (cf. Table 1) were purchased from Peptides & elephants GmbH (Potsdam, Germany) and GenicBio Limited (Shanghai, China). All peptides were purified by HPLC (>95% purity) and analyzed by mass-spectrometry. Prior to all experiments, to remove residual TFA, the peptides were dialyzed against an appropriate buffer (dialysis tubing Spectra/Por^®^ 7 with 1 K MWCO; Spectrum Labs, USA).

**Table 1:** Peptide nomenclature and experimental conditions of the NMR, ITC and fluorescence experiments

Number in the scheme (Fig. 1)	Synthetic peptides (name; residues; sequence)	NMR (buffer; pH; temperature; stoichiometry)	ITC	Fluorescence (buffer; pH; stoichiometry)	Comments
1	V5p1 (long); 696–729; S696G; GSHRGWEILRQNTLGHLNLGLNLSEGDGEEVYHF	CaM buffer^a^; pH 7.0; 25°C; 1:0–1:4	N/A	N/A	Characterized in [1] 43% sequence identity with the corresponding TRPV6 fragment
1	V5p1s (short); 696–712; S696G; GSHRGWEILRQNTLGHL	CaM buffer; pH 7.0; 35°C; 1:0–1:4.8	CaM buffer; pH 7.0; 25°C	CaM buffer; pH 7.0; 1:0–1:2	W701
1	V6p1; 690–716; R690G;GSSANWERLRQGTLRRDLRGIINRGLE	CaM buffer; pH 7.0; 35°C; 1:0–1:2.6	CaM buffer; pH 7.0; 25°C	CaM buffer; pH 7.0; 1:0–1:2	W695
2	V5p2; 591–612; ELWRAQVVATTVMLERKLPRCL	H_2_O+bMeOH; pH 7.0; 35°C; 1:0–1:1.5	N/A	H_2_O+bMeOH; pH 7.0; 1:0–1:2	W593 86% sequence identity with the corresponding TRPV6 fragment
2	V5p2n; 591–612; C611SELWRAQVVATTVMLERKLPRSL	H_2_O+bMeOH; pH 7.0; 35°C; 1:0–1:1.5	N/A	H_2_O+bMeOH; pH 7.0; 1:0–1:2	W593
3	V5p3; 401–428; L401G;GLEI PDIFRVGASRYFGKTILGGPFHVI	AmAc buffer^b^; pH 5.0; 35°C; 1:0–1:2.8	N/A	N/A	68% sequence identity with the corresponding TRPV6 fragment
3	V6p3; 401–428; L401G;GVEVPDIFRMGVTRFFGQTILGGPFHVL	AmAc buffer; pH 5.0; 35°C; 1:0–1:2.0	N/A	N/A	
4	V5p4; 310–330; C330S;GQTPVKELVSFKWNKYGRPYFS	CaM buffer; pH 7.0; 35°C; 1:0–1:3.0	N/A	CaM buffer; pH 7.0; 1:0–1:2	W322 81% sequence identity with the corresponding TRPV6 fragment
5	V5p5; 133–154; VRALLTRRASVSARATGTAFRR	CaM buffer; pH 7.0; 35°C; 1:0–1:3.0	N/A	N/A	68% sequence identity with the corresponding TRPV6 fragment

^a^CaM buffer is 20 mM Tris, 50 mM KCl, 10 mM CaCl_2_ pH 7.0

^b^AmAc buffer is 5 mM ammonium acetate, 10 mM CaCl_2_ pH 5.0

### Protein expression and purification

Recombinant *Xenopus laevis* calmodulin (identical to mammalian) was expressed in *E. coli* AR58 cells carrying the pTNcoI2 plasmid (kind gift of Dr. C. Klee, NIH, Bethesda, MD, USA). Expression was induced by a temperature shift from 30 to 42°C and 3–4 h after induction cells were harvested and lysed. Calmodulin was purified by weak anion exchange (DEAE, GE Lifesciences) and affinity chromatography (Phenylsepharose, GE Lifesciences). Purified protein was dialyzed against 1/100 of KCl 50 mM, CaCl_2_ 10 mM, Tris 20 mM pH 7.0, freeze-dried and stored at −20°C. Unless specified differently, all chemicals were obtained from Sigma-Aldrich, USA). Uniformly ^13^C and/or ^15^N-labelled samples were produced by growing the cells in M9 minimal medium containing 4 g/L ^13^C_6_-glucose and/or 1 g/L ^15^N-ammonium chloride (Buchem BV, Apeldoorn, The Netherlands) as the sole source of carbon and/or nitrogen, respectively.

### NMR spectroscopy

For the titration by synthetic peptides, ^15^N-labeled CaM samples with concentration 0.3–0.7 mM were used. CaM and peptides were dissolved in either KCl 50 mM, CaCl_2_ 10 mM, Tris 20 mM pH 7.0 (V5p1s, V6p1, V5p4, V5p5), or 10 mM CaCl_2_, 10 mM ammonium acetate buffer pH 5.0 (V5p3 and V6p3), or MilliQ water with 10 mM 2-mercaptoethanol (V5p2 and V5p2n). Notably, upon dissolving the peptide V5p2 in any buffer other than water precipitate was formed.

The experimental conditions for each peptide are summarized in Table 1.

The binding of target peptides to CaM was monitored by 2D ^15^N-^1^H-HSQC spectra. The combination of 1D ^15^N-filtered and 1D ^15^N-^1^H-HSQC experiments was used to assess the ratio of bound and non-bound peptides. ^15^N-T_1rho_ experiments were used to estimate the transverse relaxation rates of the CaM resonances during the titration. For sequential assignment of CaM 3D HNCACB and CBCA(CO)NH experiments were performed.

All spectra were recorded at 308 K on Varian Inova 800 MHz or Bruker Avance III 600 MHz spectrometers, each equipped with a cryogenic probe.

All NMR spectra were processed using the NMRpipe suite [13] and analyzed by the program CcpNmr Analysis [14].

### Fluorescence spectroscopy

Fluorescence measurements were performed using a Varian Cary Eclipse fluorescence spectrophotometer (Agilent Technologies, USA), with emission and excitation slit width set to 5 nm. Tryptophan excitation wavelength was set to 282 nm and emission spectra were recorded in the range 295–405 nm. Each spectrum was recorded three times and averaged. Fluorescence intensity contribution from CaM and background intensity from buffer and cuvettes were subtracted for each spectrum. The recorded emission intensities were corrected for dilution effects. All measurements were carried out at room temperature. CaM and peptides stocks were prepared in 50 mM KCl, 10 mM CaCl_2_ 20 mM Tris pH 7.0 buffer. The concentration of CaM in the samples was 5 μM, peptides were added up to a CaM: peptide 2:1 molar ratio. For each titration point, the samples were kept at room temperature for 30 min prior to measurements to ensure that the equilibrium was reached.

### Isothermal titration calorimetry (ITC)

ITC samples were prepared by dissolving CaM and peptides in 50 mM KCl, 10 mM CaCl_2_, 20 mM Tris pH 7.0 buffer. The concentration of CaM in the cell was 15–20 μM, the concentration of peptides in the syringe exceeded the CaM concentration by 15–20 times. Protein concentrations were determined using a Nanodrop spectrophotometer ND-1000 (Isogen Life Science). ITC experiments were carried out using an ITC_200_ device (MicroCal Inc., Northampton, MA, USA). After thermal equilibration at 298 K, thirty-nine 1 μl serial injections were performed at 500 rpm stirring speed with an injection spacing of 4 min. To correct the experimental binding isotherm for background heat effects, we also titrated peptide into buffer. All measurements were performed at least three times to determine the optimal experimental conditions with respect to signal-to-noise ratio. In all cases, experiments were consistent and reproducible. ITC data analysis was performed using the Origin 7.0 software supplied by the manufacturer of the ITC. All data were analyzed with “one binding site model” and the “sequential binding site model” implemented in this software package.

## Results

### Identification of CaM binding sites and peptide synthesis

We first identified potential CaM binding sites within TRPV5/6 by in silico prediction using the calmodulin target database server [15]. Two CaM binding sites at the TRPV5 C-terminus and one binding site at the TRPV6 C-terminus had been already reported [12, 16] and were correctly identified by the server. In total, five predicted binding sites (Fig. 1 and Supplementary Fig. 1s) were chosen for in vitro characterization and the corresponding synthetic human TRPV5/6 peptides were obtained. The details regarding these five peptides are summarized in Table 1.

### Binding of CaM to the TRPV5 C-terminal peptide 1

In our previous work [12] we studied a long version of the C-terminal TRPV5 peptide 1 (V5p1, residues 696–729 in human TRPV5). Subsequent pull-down assays showed that C-terminal deletion of 17 amino acids (yielding V5p1s, residues 696–712 in human TRPV5) did not affect TRPV5 binding to CaM-Sepharose (S. Verkaart, private communication), so we first decided to confirm this observation in vitro using various other biophysical assays.

**Fig. 2:**
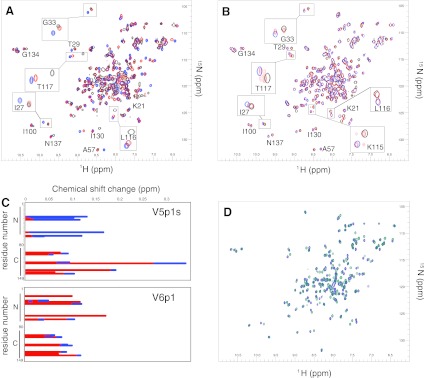
Overlaid ^15^N-^1^H-HSQC CaM-peptide NMR spectra and data analysis. Five to ten contours were deliberately spaced closely to allow for proper overlaying of the spectra and emphasis of the changes. **a** CaM-V5p1s spectra at CaM:V5p1s 1:0 (*black*), 1:1 (*red*) and 1:2 (*blue*) molar ratio. **b** CaM-V6p1 spectra at CaM:V6p1 1:0 (*black*), 1:1 (*red*) and 1:2 (*blue*) molar ratio. **c** Chemical shifts induced by the peptide as a function of CaM residue number at CaM-peptide 1:1 (*red*) and 1:2 (*blue*) molar ratio. **d** CaM-V5p1s (*dark blue*) and CaM-V6p1 (teal) spectra at 1:2 CaM:peptide molar ratio

High-resolution NMR-spectroscopy provides for a detailed residue-specific assessment of CaM-peptide interactions. Three ^15^N-^1^H-HSQC spectra of the complexes of CaM with V5p1s at molar ratios CaM:peptide 1:0/1:1/1:2 are displayed superimposed in Fig. 2a. Notably, for several residues the peak positions at 1:2 molar ratio (blue spectrum) are distinct from those at 1:1 molar ratio (red spectrum), which implies two binding events, as was also observed for the longer peptide.

Using triple-resonance heteronuclear NMR experiments and the previously published assignment of CaM (BMRB entry 547), we rapidly established the backbone assignment of CaM under the current conditions. Based on this CaM sequential assignment, we determined that mainly the residues from C-domain of CaM (81–149) are involved in the first binding event (Fig. 2c). Interestingly, increasing the peptide concentration up to 1:2 molar ratio mainly caused a shift of the resonances in the spectrum for the residues from the N-domain of CaM (1–79). Further addition of the peptide caused only minor changes including shifting of the equilibrium towards the 1:2 complex (data not shown).

**Fig. 3:**
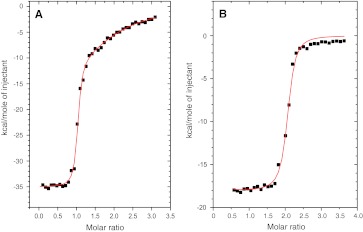
ITC data for the titration of CaM by V5p1s (**a**) and V6p1 (**b**). Integrated heat pulses and curves (*red*) fitted using “sequential binding sites model” are shown for each peptide. The resulting thermodynamic parameters are listed in Table 2

We next tested V5p1s peptide binding using ITC. Figure 3a shows the integrated heat pulses when titrating V5p1s to CaM, together with a fitted curve (red). The best fitting results were obtained using the “sequential binding sites model”. This model accounts for the non-independence of the individual peptide-binding events and suggests the number of binding sites before the fitting, which in this case was set to two. In accordance with the NMR data, the ITC results indicate two distinct events with macroscopic binding constants difference of approximately three orders of magnitude (cf. Table 2).

**Table 2:** ITC data for CaM titration by V5p1s and V6p1

Peptide	K_1_ (M^−1^)	K_2_ (M^−1^)	ΔH1 (kcal mol^−1^)	ΔH2 (kcal mol^−1^)	ΔS1 (cal mol^−1^ deg^−1^)	ΔS2 (cal mol^−1^ deg^−1^)	N
V5p1s^a^	9.2 (±4.1) × 10^6^	2.9 (±1.9) × 10^4^	−38.0 ± 0.6	−25.2 ± 0.9	−95.3	−64.1	2
V6p1^a^	2.2 (±1.7) × 10^6^	5.2 (±1.5) × 10^6^	−17.5 ± 0.9	−19.0 ± 0.8	−29.6	−33.0	2
V6p1^b^	1.3 (±0.3) × 10^7^	–	−18.0 ± 1.9	–	−27.7	–	2.12 ± 0.01

^a^Sequential binding sites model; N is fixed to the value 2

^b^One binding site model; N is allowed to vary

**Fig. 4:**
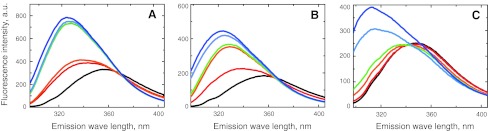
Tryptophan fluorescence spectra of the peptides V5p1s (**a**), V6p1 (**b**) and V5p2 (**c**) in response to titration by CaM. Peptide:CaM ratios 1:0 (*black*), 1:0.5 (*red*), 1:0.75 (*orange*), 1:1 (*green*), 1:1.5 (*light-blue*) and 1:2 (*dark blue*)

The V5p1s peptide contains a single tryptophan residue (W701) that has been implicated in direct interaction with CaM [12]. The steady state tryptophan fluorescence of W701 was monitored upon addition of CaM (Fig. 4a). A clear blue-shift of the emission maximum upon CaM-peptide complex formation was observed, consistent with a more hydrophobic environment of the tryptophan upon binding CaM. The extent of this blue-shift was dependent on the amount of CaM added, with the most hydrophobic environment (λ_max_ ~ 324 nm) at a peptide:CaM ratio of 1:2, which would correspond to a state in which all peptide is predominantly bound to the high-affinity CaM site.

### Binding of CaM to the TRPV6 C-terminal peptide 1

Close homologues TRPV5 and TRPV6 share 75% overall amino acid sequence identity and have been shown to form heteromeric complexes [17]. Previous investigations [9] showed that the final ~30 amino acids of TRPV6 contain a CaM binding site and that calcium-dependent channel inactivation is enabled by CaM binding to this fragment. These distal C-terminal CaM binding sites of TRPV5 and TRPV6 channels, however, have only ~43% sequence identity. This raised the question whether CaM-binding to these peptide regions occurs in a similar fashion in TRPV5 and TRPV6.

Figure 2b displays the ^15^N-^1^H-HSQC spectra of CaM upon titration by the V6p1 peptide. As also observed for V5p1s, for many CaM residues two peaks of equal intensity are observed at CaM:peptide ratio 1:1 (red spectrum). One of these peaks corresponds to free Ca^2+^/CaM, the second one—to the peptide-bound form. For the CaM residues K115 and T117 there are four peaks present. Upon further addition of V6p1 peptide up to 1:2 molar ratio (blue spectrum), all double peaks collapse into single ones positioned exactly where the peptide-bound peak originally appeared. Remarkably, in sharp contrast to V5p1s residues from both domains of CaM are involved in the first and the second binding events of V6p1 (Fig. 2c). The state of the peptide was also monitored by 1D ^15^N-filtered experiments (Supplementary Fig. 2s), which clearly established the 1:2 CaM:V6p1 binding stoichiometry.

The spectra of CaM with V5p1s and V6p1 at a molar ratio of 1:2 are displayed overlaid in Fig. 2d. The spectra are significantly distinct from each other, thus suggesting a differential organization of the two complexes.

The ITC data for the titration of CaM by the V6p1 peptide is shown in Fig. 3b. Again, the fitting was performed using the “sequential binding sites model”, in order to compare the data with those obtained for the V5p1s peptide. For the V6p1 peptide, both binding events have indistinguishable macroscopic binding constants, which are similar in magnitude to the binding constant of the high-affinity V5p1s binding event (cf. Fig. 3a; Table 2). Fitting the ITC data using the “one binding site model” (fitted data not shown) resulted in very similar values as obtained using the “sequential binding sites” model (cf. Table 2). Notably, while the thermodynamic parameters are essentially the same between the two models, this second fitting confirmed the 1:2 stoichiometry of the CaM to V6p1 binding.

Analogous to the V5p1s peptide, the V6p1 peptide also contains a tryptophan residue (W695). Tryptophan fluorescence spectra of V6p1 in the absence and the presence of different amounts of Ca^2+^/CaM are displayed in the Fig. 4b. Similar to V5p1s (Fig. 4a), we observed a blue-shift in emission maximum upon CaM-peptide complex formation. However, for V6p1 there is a gradual change of the emission maximum from 358 to 324 nm upon addition of CaM.

### Binding of CaM to the C-terminal peptide 2

The sequence corresponding to CaM binding site 2 (V5p2; cf. Table 1; residues 591–612 in human TRPV5) located at the beginning of the C-terminus is highly conserved among all TRP channels (see Supplementary Fig. 1s). It is known that the region flanking (or even overlapping with) this CaM binding site binds other molecules, including phosphatidylinositol-4,5-bisphosphate, Rab11a, 80K-H, BSPRY, S100A10/annexin 2 complex, and NHERF4 [18].

In vitro binding of CaM to the peptide corresponding to residues 587–616 of TRPV5 was previously established using fluorescence spectroscopy [16] and the equilibrium dissociation constant of peptide/Ca^2+^-CaM complex was estimated to be 0.31 ± 0.02 μM. Holakovska et al. modeled the structure of the complex based on known structures of CaM with peptides at canonical 1:1 molar ratio. Since there was no experimental data about the stoichiometry of binding of this peptide to calmodulin and given our unexpected results for the V5/6p1 peptides, we decided to check the binding of this proximal C-terminal fragment with calmodulin for possible unusual properties.

**Fig. 5:**
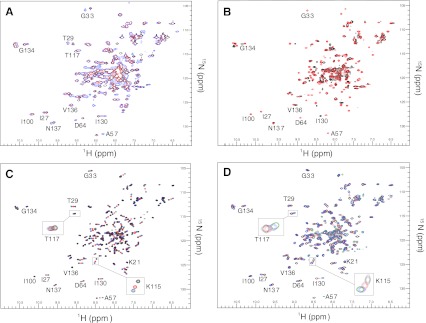
^15^N-^1^H-HSQC CaM-peptide NMR spectra and data analysis. Five to ten contours were deliberately spaced closely to allow for proper overlaying of the spectra and emphasis of the changes. **a** CaM-V5p2 spectra at CaM:V5p1s 1:0 (*black*), 1:0.75 (*red*) and 1:1.5 (*blue*) molar ratio. **b** CaM-V5p3 spectra at CaM:V5p3 1:0 (*black*) and 1:1 (*red*) molar ratio. **c** CaM-V5p4 spectra at CaM:V5p4 1:0 (*black*), 1:1 (*red*), 1:2 (*blue*) and 1:3 (*green*) molar ratio. **d** CaM-V5p5 spectra at CaM:V5p5 1:0 (*black*), 1:1 (*red*), 1:2 (*blue*) and 1:3 (*green*) molar ratio

^15^N-^1^H-HSQC spectra of the complexes of CaM with V5p2 at molar ratios 1:0 (black), 1:0.75 (red) and 1:1.5 (blue) are presented in Fig. 5a. At the concentrations desired for NMR spectroscopy, V5p2 was only soluble in H_2_O, and thus NMR samples were prepared in 95:5 v:v H_2_O/D_2_O with 10 mM 2-mercaptoethanol.

At CaM:V5p2 1:0.75 molar ratio, a significant number of the CaM peaks are doubled and for some peaks substantial line broadening is observed. At 1:1.5 molar ratio, many peaks are significantly broadened, some even beyond detection, whereas for other residues multiple peaks corresponding to bound CaM are observed. To check for potential aggregation induced by the peptide, we recorded ^15^N-T_1rho_ (spin–lattice relaxation time in the rotating frame) experiments which directly confer information regarding the rotational diffusion of the molecule and hence the oligomeric state. Using the envelope of the well-dispersed, upfield-shifted amide resonances, we obtained T_1rho_ values of ~100 ms for free Ca^2+^/CaM which decreased to ~80 ms for CaM in the 1:1.5 complex. These values are consistent with a (predominantly) monomeric state of CaM. Notably, the upfield shifted resonances correspond to residues from both domains of CaM, so the observed broadening of the ^15^N-^1^H-HSQC cross peaks is therefore most likely caused by conformational exchange. The ^15^N-^1^H-HSQC spectra of CaM titrated by the V5p2n peptide featuring a C611S mutation (to prevent possible disulfide bonds formation) is displayed in Supplementary Fig. 3s. Analogous to V5p2, at CaM:V5p2n 1.0:1.5 molar ratio a significant loss of signal is observed and an intermediate exchange regime is retained (cf. Fig. 5a). Hence, we exclude disulfide bond formation to be responsible for the major differences between V5p2/V5p2n and the other peptides.

As for the V5p1s and V6p1 peptides, we recorded tryptophan fluorescence spectra for the V5p2 peptide in the absence and in the presence of different amounts of Ca^2+^/CaM, employing its single tryptophan residue (W593) (Fig. 4c). A blue-shift in emission maximum was again observed upon addition of CaM, but it only became noticeable at CaM:peptide 0.75:1 molar ratio (shift from 350 to 337 nm). Further addition of CaM led to the emission maximum shift to 312 nm.

Due to the limited solubility of the V5p2 peptide we were not able to obtain reproducible ITC data for its interaction with CaM.

### Binding of CaM to the transmembrane peptide 3

The loop between transmembrane helices 2 and 3 (residues 408–418, numbering for mouse TRPV6; TM23-loop) has been shown to significantly affect the kinetics of TRPV5 and TRPV6 channel inactivation [19]. TRPV6 to TRPV5 mutations of residues in this loop, i.e. L409V, V411A and T412S in TRPV6, resulted in the conversion of the TRPV6 inactivation pattern to that of TRPV5. Interestingly, the TM23-loop was predicted to bind CaM by our in silico analysis and hence we decided to also check its CaM binding properties.

Peptides corresponding to the TM23-loop of TRPV5 and TRPV6 (V5p3 and V6p3, respectively) were only soluble at pH 5.0 and therefore their binding with Ca^2+^/CaM was studied in 10 mM CaCl_2_, 10 mM ammonium acetate buffer pH 5.0. Figure 5b displays the ^15^N-^1^H-HSQC spectra of the complexes of CaM with V5p3 at 1:0 and 1:1 molar ratio. Addition of more peptide did not cause any further change (supplementary Fig. 4sA). Residues of both CaM domains were involved in the binding process, demonstrating a classical 1:1 binding mode. For the titration of CaM by V6p3 similar results were obtained (supplementary Fig. 4sB). The binding patterns observed for CaM in the two complexes were also very similar (supplementary Fig. 4sC). Low pH (5.0) might influence CaM-peptide interactions but we have not studied the effect of pH in details. As both peptides contain neither tryptophan, nor tyrosine residues, fluorescence measurements were not possible using the native peptides. Unfortunately, solubility problems also prevented the analysis of these peptides using ITC.

### Binding of CaM to the N-terminal peptides 4 and 5

The in silico analysis also identified the sequences corresponding to human TRPV5 residues 133–154 and 310–330 as potential CaM interaction peptides (denoted as V5p5 and V5p4, respectively, cf. Table 1). Figure 5c and d shows ^15^N-^1^H-HSQC spectra of ^15^N-labelled CaM recorded for varying molar ratios of V5p4 and V5p5 peptides, respectively. In both cases the gradual shift of the affected resonances in response to increasing amounts of peptide indicates that the complex is in fast exchange on the NMR timescale, which is indicative for a relatively weak affinity. The selective nature of the shifting residues however, indicates that these binding events are specific. Notably, shifting of several resonances in the spectra of CaM titration by V5p4 follows a curved trajectory, which indicates that multiple processes take place during V5p4 binding to CaM.

The V5p4 peptide contains a single tryptophan residue (W322) and could be studied by fluorescence spectroscopy. It behaves in much the same way as described above for the peptides V5p1s, V6p1 and V5p2, demonstrating a blue shift upon addition of calmodulin (supplementary Fig. 5s).

## Discussion

### Interactions of calmodulin with the C-terminal peptides

We previously characterized the long version of V5p1 peptide corresponding to the distal C-terminal CaM binding sequence in TRPV5 (residues 696–729 in human TRPV5) and showed that CaM binding to this region is crucial for calcium-dependent channel inactivation. Disruption of the CaM binding site leads to a constitutively open channel [12]. Here, we established that deletion of the final 17 amino acids does not alter the CaM binding properties of this region and a shorter version of the peptide (V5p1s; residues 696–712 of human TRPV5) proved better amenable to biophysical studies. Indeed, ^15^N-^1^H-HSQC spectra of CaM titrated by the short peptide V5p1s (Fig. 2a) are highly similar to the spectra of CaM titrated by the long version of the peptide [12]. As observed for the V5p1 long peptide, a 1:2 CaM:peptide binding stochiometry was also observed for V5p1s. This stochiometry was also confirmed by the fluorescence data (Fig. 4a) and the ITC data (Fig. 3a) that displays two distinct binding events with K_a_ values of 9.2 (±4.1) × 10^6^ M^−1^ and 2.9 (±1.9) × 10^4^ M^−1^, respectively (Table 2).

The CaM-V5p1s complex allowed for the identification of the CaM resonances in the ^15^N-^1^H-HSQC spectra and hence identification of the residues involved in the binding. Surprisingly, the first, high-affinity binding event mostly involves the residues in the C-terminal domain of CaM, whereas the second event affects mostly the residues of the N-domain of CaM (Fig. 2c).

The TRPV6 is a close homolog of TRPV5, yet the binding of V6p1 peptide displays remarkable differences. The ^15^N-^1^H-HSQC spectra recorded at varying stoichiometries again indicate the formation of a 1:2 CaM:peptide complex. However, in contrast to V5p1s, in the CaM-V6p1 spectra only two CaM peak positions are observed for each residue. These correspond to the free Ca^2+^/CaM and peptide-bound CaM conformations. Exception to this are the residues K115 and T117 that display four peaks at a 1:1 stochiometry indicating involvement of these residues in a complex equilibrium and sensitivity to both binding events. 1D ^15^N-filtered experiments (supplementary Fig. 2s) confirmed 1:2 binding stoichiometry as well as the ITC data (Fig. 3b) when fitted by the “one binding site model”.

The binding of the V6p1 peptide to CaM is different compared to the V5p1s binding. In sharp contrast to V5p1s, both V6p1 binding events have nearly identical affinities (cf. Fig. 3 and Table 2). Moreover, while V5p1s demonstrates two significantly different binding events, with the C-domain of CaM involved in the first, high-affinity event, and N-domain of CaM in the second, lower-affinity one, binding of V6p1 involves both domains of CaM to a similar extent (Fig. 2c). Comparison of the ^15^N-^1^H-HSQC spectra of the 1:2 complexes of CaM with V5p1s and V6p1 (Fig. 2d) also implies that the two complexes have a different structural organization.

TRPV5 and TRPV6 both can form functional homo- or heteromeric complexes with different properties [17]. It is tempting to speculate that the differential binding of CaM to the C-terminal regions of these channels is the molecular cause of some of their functional differences.

The V5p2 peptide, corresponding to the proximal CaM binding site 2 (Fig. 1), demonstrates rather remarkable behavior: while being soluble in water, it tends to aggregate in aqueous buffer solutions and causes dramatic line broadening in the CaM NMR spectra. To exclude the formation of disulfide bridges by the single free cysteine in the V5p2 peptide as cause of this behavior, we used substantial amounts of 2-mercaptoethanol (10 mM) and tested the same peptide where the cysteine residue was substituted with a serine (V5p2n). Neither of these two conditions appears to alter the observed behavior and the self-aggregation of the peptide may be caused by its inherent propensity. Indeed, using the TANGO algorithm [20] the VVATTVML segment of V5p2 yielded a β-aggregation score comparable to that of β-amyloid peptides. Interestingly, mutation of the tryptophan residue, which is likely to be crucial for CaM binding, to alanine (W593A) led to the formation of non-functional channels (S. Verkaart, private communication), clearly also implicating the participation of this region in the channel regulation.

Unfortunately, we were not able to obtain reproducible ITC data for V5p2 interactions with CaM and hence confirm the K_d_ value 0.31 ± 0.02 μM measured in [16] using steady-state fluorescence anisotropy measurements of fluoresceinisothiocyanate labeled peptides.

### Interactions of calmodulin with the transmembrane and N-terminal peptides

We have demonstrated that the V5p3 and V6p3 peptides containing the TM23-loop bind CaM in vitro in a 1:1 canonical manner. These peptides share 68% sequence similarity and have fifteen hydrophobic/positively charged/bulky amino acids (I, L, V, F, Y, R), which together form canonical CaM binding sites [15]. However, functionally the TM23-loop is poorly characterized but has been implicated in channel inactivation [19]. Amino acids M410, V412 and T413 (numbering for human TRPV6), which were shown to be responsible for fast TRPV6 inactivation [19], are all situated inside the CaM-binding sequence and it is likely that CaM binding to this fragment provides the molecular switch for this inactivation process.

N-terminal peptides V5p4 and V5p5 also bind CaM in vitro as shown by NMR (V5p4 and V5p5) and fluorescence spectroscopy (V5p4). The V5p4 and V5p5 peptides demonstrate weak but specific binding to CaM, in line with the results for CaM-mediated interactions observed before [8]. Both peptide sequences are well conserved in eukaryotes and share 81 and 68% sequence similarity, respectively. There are several serine and threonine residues within these two CaM-binding sites, which can be phosphorylated and thus potentially could be involved in regulation, analogously to the regulatory effects observed in the distal C-terminal region of the TRPV5/6 channels [12]. Clearly, more functional data is needed to reveal the full role of CaM binding to TRPV5/6. Unfortunately, the multiple CaM interaction sites in TRPV5/6 complicate the assessment of in vivo interactions and their functional consequences.

## Conclusion

We identified and characterized in vitro five CaM binding sites in the TRPV5/6 channels. All the five sites bind calmodulin but display diversity in their binding modes, binding stoichiometries and binding affinities. Together, they may allow for a complicated and finely tuned mechanism of TRPV5/6 channel (in)activation in response to Ca^2+^, in analogy to similar effects observed for the voltage-gated Ca_v_1-2 channels.

## Electronic supplementary material

Below is the link to the electronic supplementary material.

Supplementary material 1 (DOC 26 kb)

Supplementary material 2 (EPS 1208 kb)

Supplementary material 3 (EPS 1330 kb)

Supplementary material 4 (EPS 1579 kb)

Supplementary material 5 (EPS 4420 kb)

Supplementary material 6 (EPS 419 kb)

## Abbreviations

CaM: Calmodulin
HSQC: Heteronuclear single quantum coherence
ITC: Isothermal titration calorimetry
TRPV5/6: Transient receptor potential vanilloid type 5/6
