# Supplementary material for: The TRPV5/6 calcium channels contain multiple calmodulin binding sites with differential binding properties
Source: J Struct Funct Genomics. 2012 Feb 22;13(2):91–100. doi: 10.1007/s10969-012-9128-4 (PMC3375010; doi:10.1007/s10969-012-9128-4)
Supplement: Supplementary file 1 — Supplementary material 1 (DOC 26 kb) [file 10969_2012_9128_MOESM1_ESM.doc]

**Supplementary**

Fig. 1s. Alignment of the TRPV5 and TRPV6 sequences for higher eukaryotes (m – mouse, r – rat, o – rabbit, h – human). Transmembrane domains are colored light yellow. Predicted CaM binding sites are underlined red.

Fig. 2s. 1D ^15^N-filtered spectra of the complexes CaM-V6p1. In the upfield region, only the signals of non-labeled V6p1 are observed. Up to CaM:peptide ratio 1:2 there is only one peak in the upfield region (~10.6 ppm) corresponding to the bound side chain amide group of the indol ring of W695 residue. The bottom right corner panel presents a fragment of ^15^N-^1^H-HSQC spectrum of ^15^N-labelled TRPV6 C-terminal fragment with bound and not bound forms indicated with asterisks. At 1:2.6 CaM:V6p1 ratio there is a peak appearing at ~10.1 ppm corresponding to non-bound peptide (Kovalevskaya et al. in Protein Expr Purif 80:28-33, 21)

Fig. 3s. ^15^N-^1^H-HSQC spectra of CaM-V5p2n at CaM:V5p2n 1:0 (black), 1:0.75 (red) and 1:1.5 (blue) molar ratio.

Fig. 4s. Overlaid ^15^N-^1^H-HSQC CaM-peptide NMR spectra and data analysis. A. CaM-V5p3 spectra at CaM:V5p3 1:1 (red) and 1:2 (blue) molar ratio. B. CaM-V6p3 spectra at CaM:V6p1 1:0 (black) and 1:1 (red) molar ratio. C. CaM-V5/6p3 spectra at CaM:V5p3 1:1 (red) and CaM:V6p3 1:1 (blue) molar ratio.

Fig. 5s. Tryptophan fluorescence spectra of the V5p4 peptide in response to titration by CaM. Peptide:CaM ratios 1:0 (black), 1:0.5 (red), 1:0.75 (orange), 1:1 (green), 1:1.5 (light-blue) and 1:2 (dark blue).
